# Nomograms predicting long-term overall survival and cancer-specific survival in head and neck squamous cell carcinoma patients

**DOI:** 10.18632/oncotarget.10595

**Published:** 2016-07-13

**Authors:** Jun Ju, Jia Wang, Chao Ma, Yun Li, Zhenyan Zhao, Tao Gao, Qianwei Ni, Moyi Sun

**Affiliations:** ^1^ State Key Laboratory of Military Stomatology & National Clinical Research Center for Oral Diseases & Shanxi Clinical Research Center for Oral Diseases, Department of Oral and Maxillofacial Surgery, School of Stomatology, Fourth Military Medical University, Xincheng, Xi'an, Shaanxi, China; ^2^ State Key Laboratory of Military Stomatology & National Clinical Research Center for Oral Diseases & Shanxi Key Laboratory of Stomatology, Department of Prosthodontics, School of Stomology, Fourth Military Medical University, Xincheng, Xi'an, Shaanxi, China

**Keywords:** head and neck squamous cell carcinoma, nomogram, overall survival, cancer-specific survival, prognosis

## Abstract

This study aimed to develop nomograms to predict long-term overall survival and cancer-specific survival in patients with head and neck squamous cell carcinoma (HNSCC). We conducted prognostic analyses and developed nomograms predicting survival outcome using HNSCC patient data collected from the Surveillance, Epidemiology and End Results (SEER) program of the National Cancer Institute. An external dataset of 219 patients was used to validate the nomograms. Of 36,179 HNSCC patients, 9,627 (26.6%) died from HNSCC and 4,229 (11.7%) died from other causes. Median follow-up was 28 months (1-107 months). Nomograms predicting overall survival (OS) and cancer-specific survival (CSS) were developed according to 10 clinicopathologic factors (age, race, sex, tumor site, tumor grade, surgery, radiotherapy and TNM stage), with concordance indexes (C-indexes) of 0.719 and 0.741, respectively. External validation C-indexes were 0.709 and 0.706 for OS and CSS, respectively. Our results suggest that we successfully developed nomograms predicting five- and eight-year HNSCC patient OS and CSS with high accuracy. These nomograms could help clinicians tailor surgical, adjuvant therapeutic and follow-up strategies to more effectively treat HNSCC patients.

## INTRODUCTION

Head and neck squamous cell carcinoma (HNSCC) is the most common malignant head and neck tumor. Global annual HNSCC incidence is more than 500,000 [1, 2] and in United States alone, annual incidence is 40,000 with 7,890 deaths reported [3]. Due to clinicopathologic heterogeneity and relatively high malignancy, three- and five-year HNSCC patient OS rates range from 54.0% to 93% and 46.2% to 82%, respectively [4, 5]. For patients with tumors located in important organs or with high-stage tumors, radical surgery is not advised. Therefore, accurate estimates of HNSCC patient prognoses based on clinicopathologic factors would help clinicians provide appropriate individual treatments. HNSCC patients are also at high risk of death from other factors such as liver diseases, secondary cancers and chemoradiotherapeutic toxicity [6, 7]. While deaths resulting from other causes (DROC) are often related to cancer-specific mortality (CSM), measuring CSS, rather than OS, will more accurately describe patient survival due to HNSCC directly.

The National Comprehensive Cancer Network (NCCN) guidelines [8] currently recommend the use of the American Joint Committee on Cancer (AJCC) Staging Manual (7^th^ edition) to predict HNSCC patient prognosis [9]. However, HNSCC patient stage, from I to IV, is only based on TNM stage. Consideration of other clinicopathologic factors like age, sex, tumor site and treatment, which are associated with HNSCC patient survival [10–13], is necessary for accurate prognostic analyses. Therefore, our prognosis predictions included age, sex, race, tumor site, surgery and radiotherapy, along with TNM stage.

Using nomograms to predict cancer prognosis is a growing trend. Compared with the AJCC Staging Manual, nomograms predict individual patient survival with higher accuracy. Nomograms are useful scoring and visualization tools that can transform multi-factorial Cox's or competing risks models into a single score sheet. Nomograms have been used to assisting surgeons in developing treatment and follow-up strategies in several cancers, including gastric cancer [14], adenoid cystic carcinoma [15] and breast cancer [16] based on a series of factors together. Nomogram development is also included in the NCCN guidelines [17]. One HNSCC study based on the Surveillance, Epidemiology and End Results (SEER) database between 2000 and 2010 applied nomograms [18], but TNM stage information was not available in the database until 2004, and this study did not include patients without surgery. We aimed to develop HNSCC nomograms predicting long-term OS and CSS based on multiple clinicopathologic factors, as well as TNM stage, to improve individual patient treatments and follow-up strategies.

## RESULTS

### Patient, tumor and follow-up characteristics

A total of 154,581 primary HNSCC patients diagnosed between 2004 and 2012 were collected from the SEER database of the National Cancer Institute. All patients were diagnosed above 15 years of age. 118,402 records were excluded due to clinicopathologic factor uncertainty or because information was obtained from a death certificate or autopsy. In total, 36,179 primary HNSCC patients were included in our study to conduct prognostic analyses and develop nomograms, and the median follow-up length was 28 months (1-107 months).

Patient ages ranged from 15 to 96 years (median, 61). Of 36,179 eligible patients, 30,371 (83.9%) were white and 27,351 (75.6%) were male. 23,696 (65.5%) tumors arose from the oral cavity or pharynx. 11,915 (32.9%) tumors were poorly or un-differentiated. T3-T4 tumors accounted for 34.5% of all tumors, and positive neck nodes and distant metastases accounted for 47.0% and 2.8%, respectively. 18,977 (52.5%) and 25,852 (71.5%) patients received surgery and radiotherapy, respectively. Of these, 9,627 (26.6%) died from HNSCC and 4,229 (11.7%) died from other causes (Table 1).

**Table 1:** Patient, tumor and follow-up characteristics

Variable	SEER Cohort (*n* = 36179)		Validation Cohort (*n* = 219)	
	No.	%	No.	%
**Age, years**				
range (ME)	15-96 (61)		27-92 (62)	
**Race**				
White	30371	83.9	0	0
Black	3972	11.0	0	0
Others	1836	5.1	209	100
**Sex**				
Female	8828	24.4	82	37.4
Male	27351	75.6	137	62.6
**Tumor site**				
Oral	23696	65.5	167	76.3
Larynx	10949	30.3	38	17.4
Nasal	882	2.4	12	5.5
Salivary	652	1.8	2	0.9
**Grade**				
I	5482	15.1	23	10.5
II	18782	51.9	108	49.3
III	11609	32.1	84	38.4
IV	306	0.8	4	1.8
**T status**				
T1	12821	35.4	46	21.0
T2	10873	30.1	59	26.9
T3	5438	15.0	82	37.4
T4	7047	19.5	32	14.6
**N status**				
N0	19173	53.0	96	43.8
N1	5680	15.7	58	26.5
N2	10389	28.7	36	16.4
N3	937	2.6	29	13.2
**M status**				
M0	35152	97.2	207	94.5
M1	1027	2.8	12	5.5
**Surgery**				
Performed	18977	52.5	209	100
None	17202	47.5	0	0
**Radiotherapy**				
Performed	25852	71.5	153	69.9
None	10327	28.5	66	30.1
**Outcome**				
Alive	22323	61.7	131	59.8
CSM	9627	26.6	68	31.1
DROP	4229	11.7	20	9.1

Abbreviation: ME, median; Others, American Indian/Alaska Native/Asian/Pacific Islander; CSM, cancer-specific mortality; DROP, death resulting from other causes.

The validation cohort included 219 primary HNSCC patients diagnosed and treated between 2000 and 2008 (Table 1). All were Chinese and received surgery. Median follow-up length was 37 months (12-130 months).

### Prognostic analyses: HNSCC patient OS and CSS

By univariate analysis, all clinicopathologic factors were statistically associated with OS. Multivariate analysis indicated that all ten factors were independent prognostic factors for HNSCC OS. (Table 2)

**Table 2:** Univariate and multivariate analyses of OS in nomogram cohort

Varible	Univariate Analysis	Multivariate Analysis	
	*P* value	HR (95%CI)	*P* value
**Age, years**	**<0.001**		
15-24		0.207 (0.128-0.335)	<0.001
25-34		0.196 (0.155-0.248)	<0.001
35-44		0.186 (0.166-0.210)	<0.001
45-54		0.232 (0.215-0.251)	<0.001
55-64		0.283 (0.263-0.304)	<0.001
65-74		0.383 (0.356-0.412)	<0.001
75-84		0.622 (0.577-0.671)	<0.001
85+		Reference	
**Race**	<0.001		
White		Reference	
Black		1.375 (1.310-1.443)	<0.001
Others		0.870 (0.803-0.942)	<0.001
**Sex**	0.002		
Female		1.063 (1.022-1.106)	<0.001
Male		Reference	
**Tumor site**	<0.001		
Oral		1.054 (0.940-1.182)	0.103
Larynx		1.122 (0.989-1.151)	0.077
Nasal		1.316 (1.133-1.529)	<0.001
Salivary		Reference	
**Differentiation Grade**	<0.001		
I		0.822 (0.687-0.984)	0.033
II		1.040 (0.874-1.236)	0.661
III		0.954 (0.802-1.135)	0.599
V		Reference	
**T status**	<0.001		
T1		0.348 (0.331-0.366)	<0.001
T2		0.522 (0.499-0.545)	<0.001
T3		0.766 (0.729-0.805)	<0.001
T4		Reference	
**N status**	<0.001		
N0		0.326 (0.310-0.343)	<0.001
N1		0.521 (0.498-0.545)	<0.001
N2		0.756 (0.719-0.794)	<0.001
N3		Reference	
**M status**	<0.001		
M0		0.434 (0.402-0.467)	<0.001
M1		Reference	
**Surgery**	<0.001		
Performed		0.632 (0.608-0.656)	<0.001
None		Reference	
**Radiotherapy**	<0.001		
Performed		0.613 (0.587-0.640)	<0.001
None		Reference	

Abbreviation: Others, American Indian/Alaska Native/Asian/Pacific Islander.

According to Gray's test and a multivariate competing risks model, all factors were independent for HNSCC CSS (*P* < 0.05) (Table 3). CSM and DROC cumulative incidence functions (CIF) were significantly higher in older patients than in younger patients. Compared to whites and other races, black patient prognoses were more directly influenced by HNSCC. When tumors were located in the salivary gland and larynx, CSM CIFs were highest and lowest, respectively. HNSCC patients with higher NM stage suffered from a worse CSS, while DROC incidence decreased. Notably, patients who received surgery or radiotherapy had worse CSS rates than those who did not receive surgery or radiotherapy.

**Table 3:** Patients five- and eight-year cumulative incidences of death in nomogram cohort

Variable	CSM			DROC		
	5-year, %	8-year, %	*P* value	5-year, %	8-year, %	*P* value
**All patients**	31.0	34.9		13.4	20.2	
**Age, years**			<0.001			<0.001
15-24	19.8	29.5		7.6	7.6	
25-34	23.8	23.8		2.6	2.6	
35-44	23.4	26.1		4.1	5.5	
45-54	28.2	31.7		7.4	11.6	
55-64	30.1	34.0		11.2	17.2	
65-74	31.9	36.7		15.5	24.9	
75-84	37.2	41.1		23.5	34.9	
85+	42.6	44.3		35.0	46.7	
**Race**			<0.001			<0.001
White	29.5	33.3		13.3	20.3	
Black	43.0	47.1		15.8	21.8	
Others	29.5	34.4		9.2	15.3	
**Sex**			<0.001			0.527
Female	32.6	36.7		13.4	19.4	
Male	30.5	34.3		13.4	20.5	
**Tumor site**			<0.001			<0.001
Oral	31.2	34.8		12.0	18.0	
Larynx	30.2	34.8		15.5	23.6	
Nasal	34.0	36.7		14.5	23.5	
Salivary	33.1	35.3		23.3	35.3	
**Differentiation Grade**			<0.001			0.283
I	20.5	24.1		13.1	21.5	
II	32.3	36.1		13.8	20.5	
III	33.9	38.0		12.7	19.1	
V	33.3	41.1		14.2	21.6	
**T status**			<0.001			0.070
T1	15.5	19.3		13.3	21.5	
T2	30.0	33.8		13.3	19.6	
T3	42.7	46.7		14.3	21.7	
T4	52.5	56.1		12.9	17.7	
**N status**			<0.001			<0.001
N0	22.8	26.9		15.5	24.2	
N1	37.8	41.4		12.2	17.4	
N2	41.2	45.2		10.4	14.5	
N3	49.8	51.2		7.7	12.4	
**M status**			<0.001			<0.001
M0	29.7	33.6		13.5	20.6	
M1	75.9	79.0		8.1	8.5	
**Surgery**			<0.001			<0.001
Performed	38.7	42.9		14.0	20.1	
None	24.1	27.7		12.8	20.4	
**Radiotherapy**			<0.001			<0.001
Performed	32.5	36.7		12.8	19.4	
None	27.3	30.4		14.8	22.4	

Abbreviation: Others, American Indian/Alaska Native/Asian/Pacific Islander.

### Nomograms predicting five- and eight-year survival

**Figure 1:**
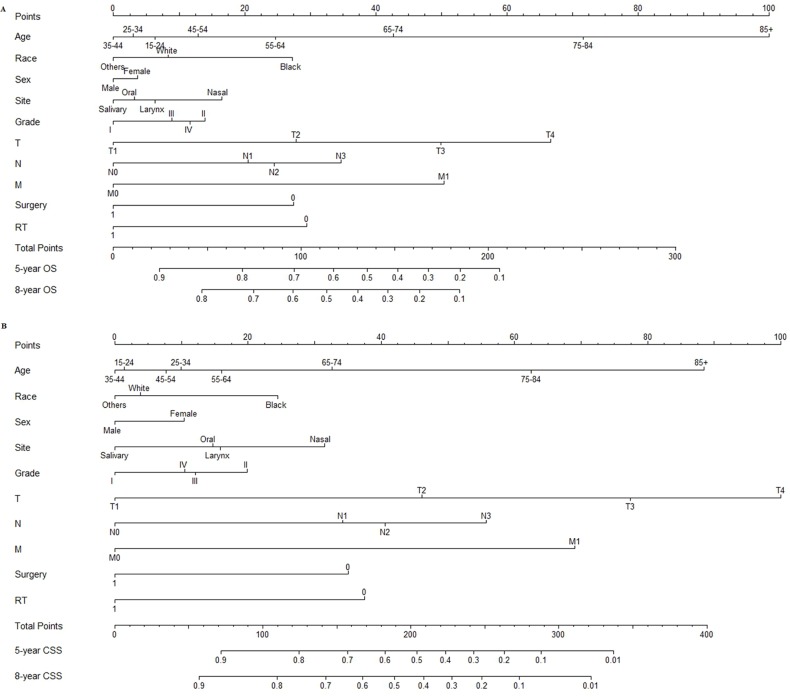
Nomograms predicting five- and eight-year OS A. and CSS B

According to the smallest AIC (26819.6) of the predictive accuracy test, all factors were selected to develop the nomogram predicting five- and eight-year OS. According to the CIF, all factors were also selected to develop the nomogram predicting five- and eight-year CSS (Figure 1).

### Nomogram validation

**Figure 2:**
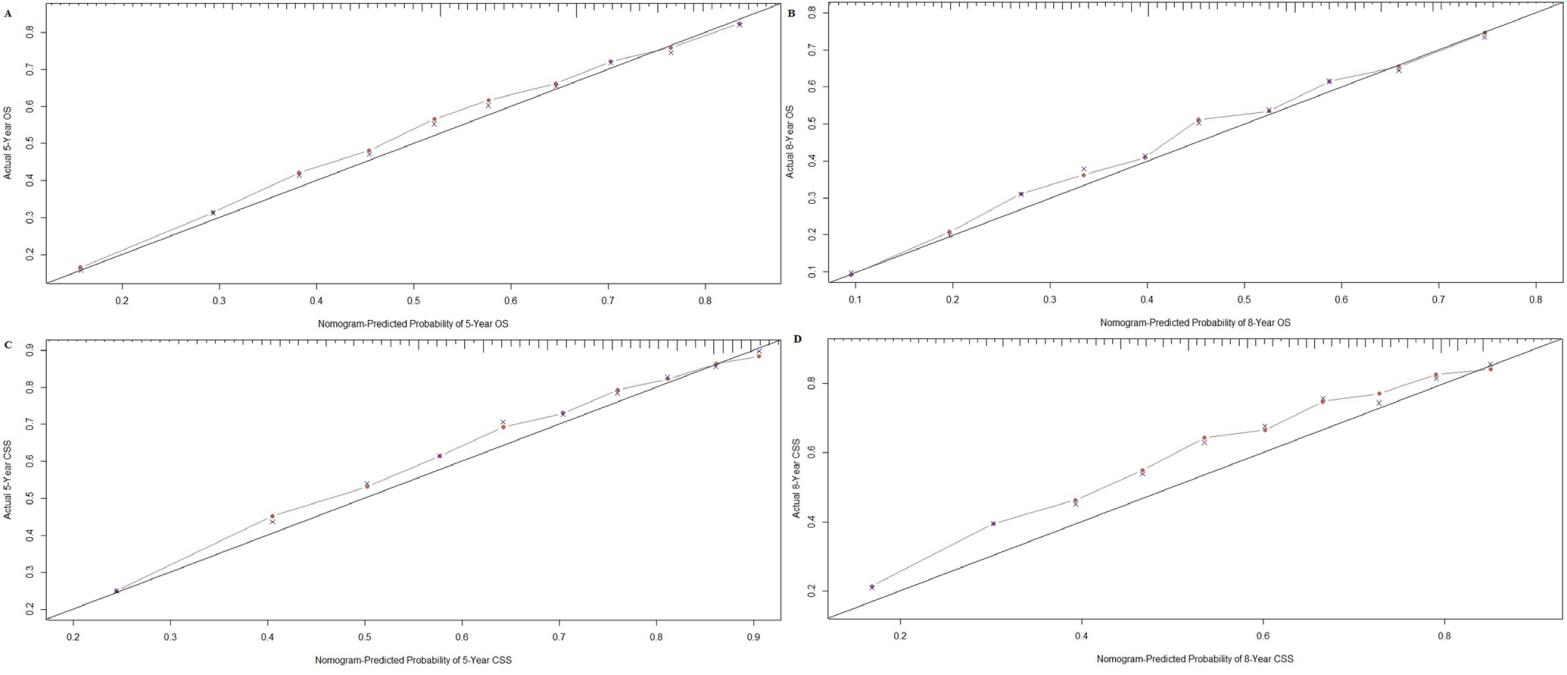
Internal calibration plot for **A.** five-and **B.** eight-year OS and **C.** five- and **D.** eight-year CSS. The 45-degree straight line represents the perfect match between the actual (y-axis) and nomogram-predicted (x-axis) survival probabilities. The nomogram cohort was divided into 10 equal groups for internal validation. A closer distance between two curves indicates higher accuracy.

**Figure 3:**
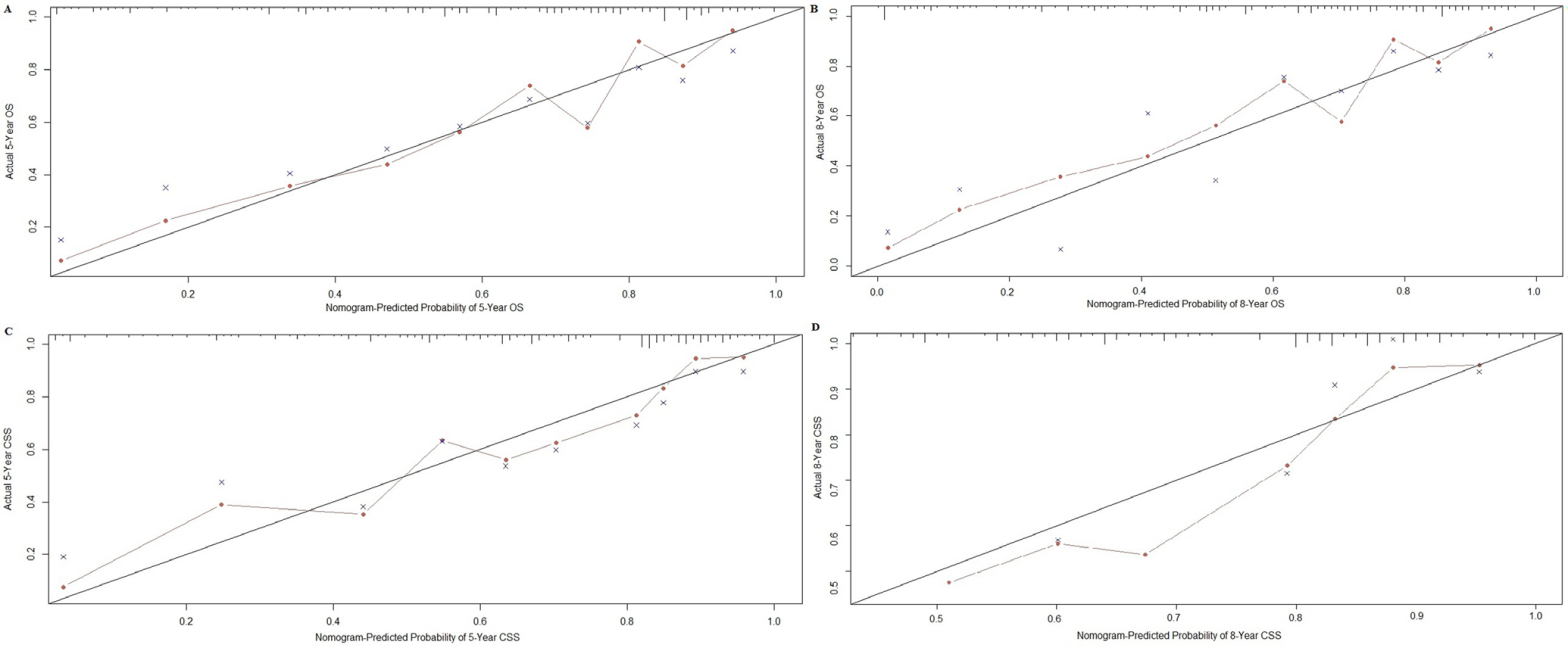
External calibration plot for **A.**, five- and **B.** eight-year OS and **C.** five- and **D.** eight-year CSS. The 45-degree straight line represents the perfect match between the actual (y-axis) and nomogram-predicted (x-axis) survival probabilities. The nomogram cohort was divided into 10 equal groups for external validation. A closer distance between two curves indicates higher accuracy.

Nomograms were validated internally and externally using bootstrap and ten-fold cross-validation methods. Internal validation *via* the training cohort showed that nomograms predicting OS and CSS were in excellent agreement with actual OS and CSS, with concordance indexes (C-indexes) of 0.719 and 0.741, respectively. External validation *via* the validation cohort showed that OS and CSS nomogram C-indexes decreased slightly to 0.709 and 0.706, respectively. Internal and external OS and CSS nomogram calibration plots showed excellent agreement between the calibration curves and the 45-degree perfect match straight lines (Figures 2 and 3).

## DISCUSSION

Due to its high incidence and CSM rates, HNSCC is an increasing public health burden. Improved strategies to identify and treat high-risk HNSCC patients are urgently needed. The decision to treat HNSCC surgically is made according to several factors, including age, pathologic features and tumor site, and a considerable number of patients (50% in our study) do not receive surgery. Radiotherapy, chemotherapy and chemoradiotherapy are frequently performed in HNSCC patients for whom surgery is not advised [19, 20]. As a large proportion of HNSCC patients do not receive surgery, it is very important to evaluate patient prognosis and accurately predict the effects of adjuvant therapy.

To ensure nomogram accuracy, we used Cox's proportional hazards regression model and the number of AIC to obtain the factors used in developing the OS nomogram. We obtained the factors used in developing the CSS nomogram *via* a competing risks model. These clinicopathologic factors were then used to develop nomograms predicting five- and eight-year HNSCC patient OS and CSS. All nomogram C-indexes were more than 0.7 and there was excellent agreement between calibration curves and 45-degree perfect match straight lines.

Using a nomogram to predict patient survival is simple. First, to include every clinicopathological factor, a vertical line should be drawn from every factor to the “Points” line in nomogram. Then, the total points value is obtained, and a vertical line should be drawn from the “Total Points” line to the survival probability line to obtain the corresponding survival. For example, consider a 55-year-old male white patient with a T3N0M0 moderate-differentiated salivary squamous cell carcinoma who underwent both surgery and radiotherapy. Using nomograms, we estimate that he has an eight-year OS of 61% and an eight-year CSS of 76%. In addition, this step can also be conducted by command “predict” of R program [21].

We also performed HNSCC prognostic analyses for OS and CSS. OS and CSS declined for patients older than 55 years. Among patients younger than 55 years old, those aged 35-44 had the best survival. Age was found to be an important prognostic factor in several studies [18, 19, 22], although the reason for this is currently unclear. The mortality rates of black HNSCC patients were higher than that of other races. A National Cancer Data Base (NCDB) study also showed that five-year survival for HNSCC patients was lower for African-Americans [23]. Accordingly we hypothesize that melanin might promote HNSCC growth in black patients, while no relevant study has been reported. Contrary to OS and CSM trends, DROC improved with increasing NM stage, indicating that NM stage influences HNSCC CSS.

Both five- and eight-year CSM of HNSCC patients who received radiotherapy (32.5% and 36.7%) were higher than those of patients without radiotherapy (27.3% and 30.4%), while DROC decreased with radiotherapy. On the contrary, radiotherapy improved HNSCC patient OS (*P* < 0.001). This may be because radiotherapy was primarily used to treat patients with higher-grade disease or those who could not receive surgery. In the training cohort, patients with a T3-T4 tumor (80.6%) or an N+ tumor (85.8%) received more radiotherapy than average (71.5%). Among patients who did not receive surgery, 87.7% had radiotherapy, as compared to only 56.5% of patients who did receive surgery. Excluding all patients treated surgically, the five- and eight-year CSMs of patients who received radiotherapy (34.4% and 39.1%) were improved compared with non-radiotherapy-treated patients (58.9% and 61.5%). However, when patients who were not treated surgically were excluded, radiotherapy did not improve CSM, but did increase DROC. Thus, radiotherapy might decrease CSM in patients with higher-grade disease and in those for whom surgery is not advised.

Our results suggest that nomograms not only accurately estimate the effects of all independent factors on survival, but also clearly estimate the prognostic values of single factors when Cox's or competing risks models fail. Although our nomograms were of high accuracy according to C-indexes and calibration plots, there are likely important independent prognostic factors that have not yet been identified by researchers or recorded by the SEER program, and these factors were not included in our study.

Our study had certain limitations. First, HNSCC TNM stage information was not available from the SEER program until 2004. The earliest record included in our study was diagnosed in that year, and we failed to predict a survival time longer than than eight years. Second, factors like human papillomavirus [24], perineural invasion [25] and chemotherapy [26] were associated with HNSCC prognosis, but were not recorded by the SEER program, and thus were not included in our nomograms. For the same reason, nomograms predicting loco-regional control or disease-free survival were not developed.

In conclusion, based on a large patient cohort from the SEER database, we conducted prognostic analyses and developed nomograms predicting five- and eight-year HNSCC patient OS and CSS with high accuracy. These nomograms could help clinicians tailor surgical, adjuvant therapeutic and follow-up strategies to more effectively treat HNSCC patients.

## MATERIALS AND METHODS

### Patient data collection

HNSCC patient data were collected from the SEER program of the National Cancer Institute (training cohort) [27] and a medical center (Department of Oral and Maxillofacial Surgery, School of Stomatology, Fourth Military Medical University, China, validation cohort). For data collected from the SEER program, initial selection criteria were as follows: The HNSCC primary tumor site was the oral cavity, pharynx, larynx, nose, nasal cavity or middle ear; malignant behavior; older than age 15; and diagnosed between 2004 and 2012. Final selection criteria were as follows: active follow-up; clinical and pathological information, including age, race, sex, tumor site, tumor grade, surgery, radiotherapy and TNM stage, were complete and definite; and data was collected not from an autopsy or death certificate. Patients were divided into several groups according to age (grouped by 10 years) ranging from 15 to 84 years, with one group of patients over 85 years of age. Race classifications included white, black and others (American Indian/Alaska Native/Asian/Pacific Islander). Tumor site classifications included salivary gland, larynx, oral cavity/pharynx and nose/nasal cavity/middle ear.

### Prognostic analyses

Prognostic analysis was conducted according to the training cohort collected from the SEER database. Ten clinicopathologic factors, including age, race, sex, tumor site, tumor grade, surgery, radiotherapy and TNM stage, were used to conduct the analyses.

### HNSCC patient OS

OS was defined as failure if a patient died, or censoring if a patient was alive at the last follow-up. OS length was defined as the time from diagnosis to failure or censoring. Log-rank test and Cox's proportional hazards regression model were used to conduct the prognostic analysis, and factors with a *P* value less than 0.2 in univariate analyses were included in the multivariate analysis to obtain independent HNSCC OS factors (*P* < 0.05) [28].

### HNSCC patient CSS

CSS was defined as failure if a patient died due to HNSCC, or censoring if a patient was alive at the last follow-up or dead due to other reasons. CSS length was defined as the time from diagnosis to failure or censoring. Gray's test and competing risks model were applied to conduct the competing risks prognostic analysis and obtain CIFs for different category groups [29]. Cutoff of time was 5 and 8 years. Categories with a *P* value less than 0.05 were considered as independent HNSCC CSS factors.

SPSS version 19.0 (SPSS, Chicago, IL, USA) was used to conduct OS prognostic analysis. R version 3.2.4 software (Institute for Statistics and Mathematics, Vienna, Austria; www.r-project.org) was used to conduct CSS prognostic analysis.

### Nomogram development

Nomograms were developed using the training cohort. Follow-up length was defined as the time from diagnosis to failure or censoring. Observed OS and CSS times were estimated by median follow-up length. Cox's proportional hazards regression model was conducted by the R software “cph” and “step” commands. Age, race, sex, tumor site, tumor grade, surgery, radiotherapy and TNM stage were used as the starting factor combination. The Akaike Information Criterion (AIC) [30] was obtained *via* the above-mentioned commands, and the combinations of factors with the smallest number of AIC were used as the models to develop nomograms predicting five- and eight-year HNSCC patient OS [31]. Independent CSS factors obtained *via* competing risks model were used to develop nomograms predicting five- and eight-year HNSCC patient CSS. R version 3.2.4 software was used to develop nomograms.

### Nomogram validation

Nomogram validations were conducted both internally (training cohort) and externally (validation cohort) by two methods, C-index and calibration plot. 1000 times bootstrapping and ten-fold cross-validation was used in internal and external validations, respectively. A C-index is an index of probability of concordance between predicted and actual situations, ranging from 0.5 to 1.0 (perfect agreement). A Calibration plot is a graph consisting of two curves, a 45-degree straight line (perfect match) and an irregular curve (calibration curve). Distance from the irregular curve to the straight line is proportional to nomogram accuracy. C-index and calibration plot were obtained by R software *via* the “rcorrcens” and “calibrate” commands, respectively.

### Ethics statement

Our study was approved by the Fourth Military Medical University Ethical Committee. Informed patient consent was not required for data released by the SEER database.
